# Supplementary material for: The interactome of intact mitochondria by cross-linking mass spectrometry provides evidence for coexisting respiratory supercomplexes
Source: Mol Cell Proteomics. 2017 Dec 8;17(2):216–32. doi: 10.1074/mcp.RA117.000470 (PMC5795388; doi:10.1074/mcp.RA117.000470)
Supplement: Supplemental Data [file supp_RA117.000470_133922_0_supp_23978_fzffwf.zip › spectra_annotation/mito_DR_spectra_annotation/202-1-4-1-8-1.pdf]

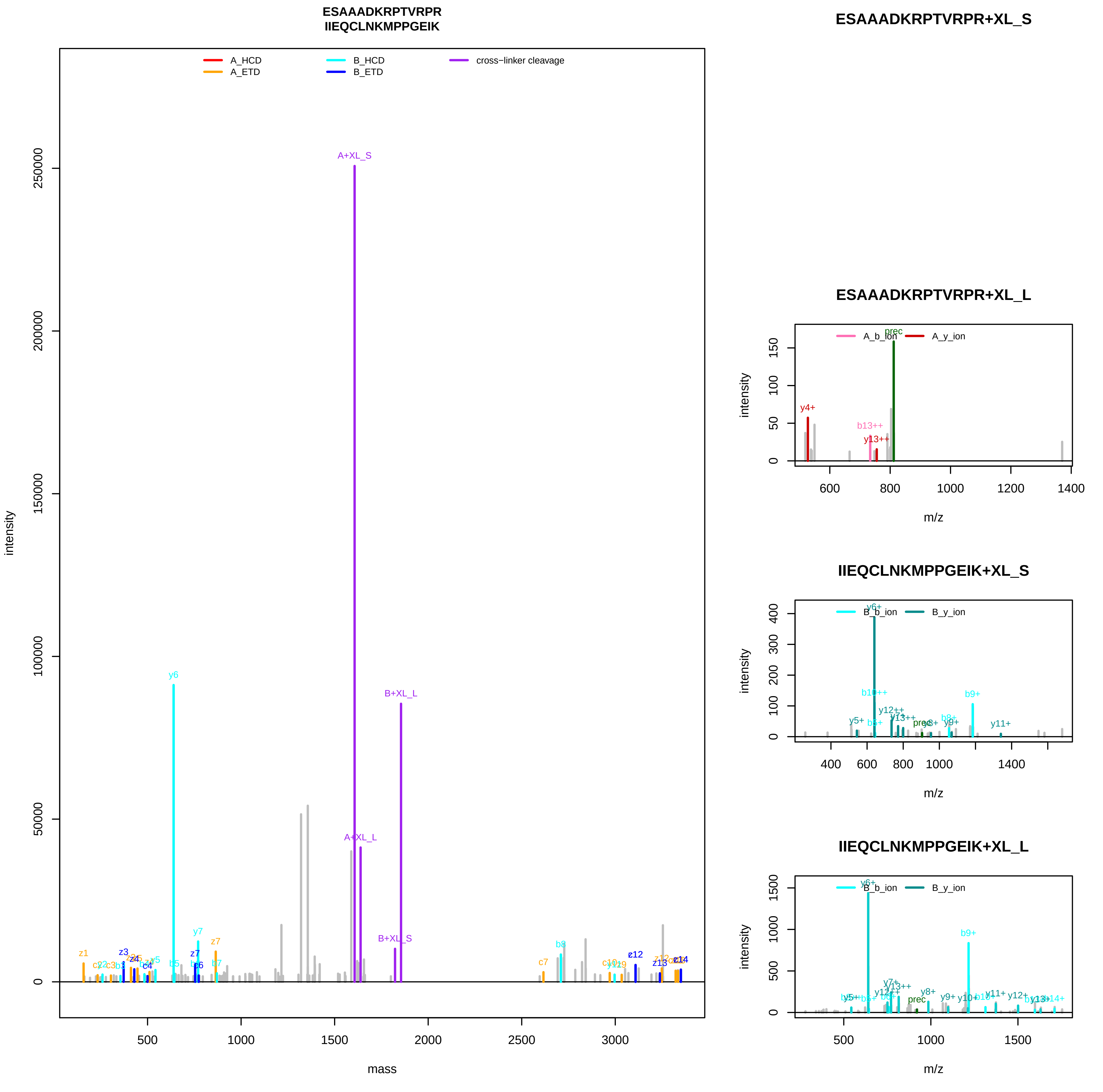

ESAAADKRPTVRPR
IIEQCLNKMPPGEIK
A_HCD
A_ETD
B_HCD
B_ETD
cross−linker cleavage
A+XL_S
B+XL_L
A+XL_L
B+XL_S
y6
y7
z7
z1
z3
z4
c4
c6
b7
c7
b8
z12
z13
c14
intensity
mass
0
50000
100000
150000
200000
250000
500
1000
1500
2000
2500
3000
ESAAADKRPTVRPR+XL_S
ESAAADKRPTVRPR+XL_L
A_b_ion
A_y_ion
prec
y4+
b13++
y13++
intensity
m/z
0
50
100
150
600
800
1000
1200
1400
IIEQCLNKMPPGEIK+XL_S
B_b_ion
B_y_ion
y6+
b10++
y12++
y13++
y5+
b9+
y11+
y9+
intensity
m/z
0
100
200
300
400
400
600
800
1000
1400
IIEQCLNKMPPGEIK+XL_L
B_b_ion
B_y_ion
y6+
b9+
y7+
y13++
y12++
y8+
prec
y9+
y10+
y11+
y12+
y5+
intensity
m/z
0
500
1000
1500
500
1000
1500
